# Broadband Cooperative Spectrum Sensing Based on Distributed Modulated Wideband Converter

**DOI:** 10.3390/s16101602

**Published:** 2016-09-28

**Authors:** Ziyong Xu, Zhi Li, Jian Li

**Affiliations:** College of Electronics and Information Engineering, Sichuan University, Chengdu 610065, China; scuxuziyong@163.com (Z.X.); lijiandz@scu.edu.cn (J.L.)

**Keywords:** cognitive radio, compressed sensing, cooperative spectrum sensing, transmission loss, distributed modulated wideband converter, frequency support

## Abstract

The modulated wideband converter (MWC) is a kind of sub-Nyquist sampling system which is developed from compressed sensing theory. It accomplishes highly accurate broadband sparse signal recovery by multichannel sub-Nyquist sampling sequences. However, when the number of sparse sub-bands becomes large, the amount of sampling channels increases proportionally. Besides, it is very hard to adjust the number of sampling channels when the sparsity changes, because its undersampling board is designed by a given sparsity. Such hardware cost and inconvenience are unacceptable in practical applications. This paper proposes a distributed modulated wideband converter (DMWC) scheme innovatively, which regards one sensor node as one sampling channel and combines MWC technology with a broadband cooperative spectrum sensing network perfectly. Being different from the MWC scheme, DMWC takes phase shift and transmission loss into account in the input terminal, which are unavoidable in practical application. Our scheme is not only able to recover the support of broadband sparse signals quickly and accurately, but also reduces the hardware cost of the single node drastically. Theoretical analysis and numerical simulations show that phase shift has no influence on the recovery of frequency support, but transmission loss degrades the recovery performance to a different extent. Nevertheless, we can increase the amount of cooperative nodes and select satisfactory nodes by a different transmission distance to improve the recovery performance. Furthermore, we can adjust the amount of cooperative nodes flexibly when the sparsity changes. It indicates DMWC is extremely effective in the broadband cooperative spectrum sensing network.

## 1. Introduction

Cognitive radio is considered as a potential technology which improves spectral efficiency drastically [1,2]. It allows the second users (SUs) to monitor the frequency ranges that are allocated to the primary users (PUs) and search the spectrum holes to access them dynamically. As is well known, spectrum sensing is a core technology in cognitive radio. There are several basic sensing methods that are widely used in spectrum sensing. For instance, energy detection is the simplest but the most noise-sensitive approach [3]. A trade-off between the signal-to-noise ratio (SNR) and a priori knowledge is considered in cyclostationary detection, which requires knowledge of the modulation type of the signal and assumes the signal exhibits cyclostationarity [4,5]. In addition, matched filter detection is the optimal approach in which all a priori knowledge of the signal is required [6,7]. However, all the above-mentioned basic approaches apply to the low frequency (LF) field.

In order to search as many spectrum holes as possible, cognitive radio must apply to the high frequency (HF) field. However, this idea is subject to the Nyquist sampling rate. Compressed sensing (CS) overcomes this obstacle perfectly. It recovers the support of the broadband signal by the sub-Nyquist sampling sequence [8,9].Then we can locate the all spectrum holes by the frequency support. However, this approach is energy detection in essence, which is unreliable in a low SNR situation. Cooperative spectrum sensing (CSS) is proposed to improve sensing robustness, but it suffers from multipath and shadow fading [10,11,12]. Furthermore, CSS can combine with distributed compressed sensing (DCS) expediently to take full advantage of sensor nodes’ spatial diversity to improve the sensing performance. The second joint sparsity model (JSM-2) of DCS demands that all sensor nodes have the same support [13], which is subsistent in the cooperative spectrum sensing scene.

Modulated wideband converter (MWC) is a kind of analog sub-Nyquist sampling system [14]. It reconstructs the broadband signal of interest accurately by the low-speed sampling sequences. It is especially fit to conduct wideband spectrum sensing, because of solving the sampling rate bottleneck in the practical deployment of cognitive radio. Its feasibility is validated by the MWC prototype circuit board [15]. However, MWC possess a troublesome fact: that the amount of sampling channels becomes very large when the sparsity is great. It is an unacceptable hardware cost for a single node. Besides, in MWC it is difficult to adjust the number of sampling channels when the sparsity changes, because its undersampling board is designed by a given sparsity.

In this paper, we propose a distributed modulated wideband converter (DMWC) scheme. It can combine undersampling technology with broadband cooperative spectrum sensing skillfully. In the sensor network, DMWC treats every single geographical sensor node as a sampling channel, which conducts stochastic mixing, low-pass filtering and low-speed sampling to the broadband signal of interest. The low-cost micro controller unit (MCU) with the low-speed A/D module is enough for sensing. However, different phase shift and transmission loss will be brought in between different channels. Even so, we only care about the accurate recovery of frequency support, not the time-domain expression. We analyze the recovery performance when there is phase shift and transmission loss between different sampling channels. Theoretical analysis and numerical simulations indicate phase shift has no influence on the recovery of frequency support, but transmission loss degrades the recovery performance to a different extent.

## 2. Cooperative Spectrum Sensing Model

**Figure 1:**
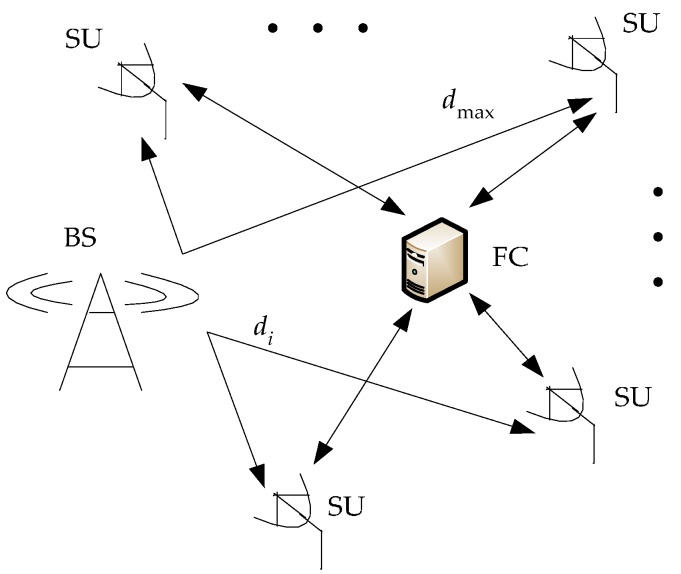
Three elements are included in a CSSN.

Energy detection is extremely sensitive to noise uncertainty and achieves poor detection performance at low SNR levels. Because of the fundamental limits, accurate detection is impossible below the SNR wall level [16,17]. Besides, reconstruction algorithms can only recover support accurately under high SNR levels, such as the orthogonal matching pursuit (OMP) algorithm [18]. To improve the performance of spectrum sensing, and take full advantage of sensor nodes’ spatial diversity to fight against fading and the hidden nodes problem, CSS based on CS is considered. The model of the cooperative spectrum sensing network (CSSN) is given by Figure 1. There are three elements in a CSSN, including a base station (BS), plenty of SUs and a fusion center (FC).

There are m SUs in the CSSN, and the distance between BS and the i-th SU is $d_{i},\;i=1,2,\ldots ,m$. The farthest transmitting distance is marked as $d_{\max}$, and the original transmitting signal of BS is x(t). Each SU knows its $d_{i}$ and geography position in the CSSN, and sends these messages with attached sampling data to the FC. For simplicity and universality, we assume our signal model is a free space propagation model, so phase shift and transmission loss in each transmission path between SU and BS can be calculated as the priori knowledge according to $d_{i}$ and the signal bandwidth. Phase shift $\theta_{i}$ can be calculated by $2\pi d_{i}/CT$, C is the signal transmission speed and T is the period of the broadband signal. The transmission loss coefficient $\alpha_{i}$ is calculated by:

(1) $$\alpha_{i}=\frac{P_{a}}{P_{t}}=\frac{G\lambda^{2}}{{\left(4\pi \right)}^{2}d_{i}{}^{2}F}\;,$$

where $P_{a}$ is the received power, $P_{t}$ is the transmitted power, G is the system gain and F denotes the system loss factor [19]. Obviously, when $\alpha_{i}$ becomes smaller, the attenuation is greater. Now, we can represent the received signal in the i-th SU as:

(2) $$x_{i}(t)=\alpha_{i}e^{j\theta_{i}}x(t)\;.$$

In the JSM-2 model of DCS, the support S of the original signal is equal to each sensor node’s support $S_{i}$. It means all nodes satisfy jointly sparse property. We apply this thinking to CSSN: each SU conducts sub-Nyquist sampling independently, and the FC collects all sampling data to complete the reconstruction uniformly. The FC communicates with each SU by a mechanism that is called the three-time handshake. We use the DMWC framework to conduct sub-Nyquist sampling in the CSSN. The only difference with MWC is that each SU just conducts one-channel sampling with a mixer, a low-pass filter (LPF) and a low-speed analog-to-digital conversion (ADC). On the one hand, we can drastically reduce the hardware cost of a single SU. On the other hand, it is easy to adjust and satisfy the number of sampling channels we need, benefiting from the fact that plenty of SUs are included in a CSSN.

## 3. Distributed Modulated Wideband Converter

**Figure 2:**
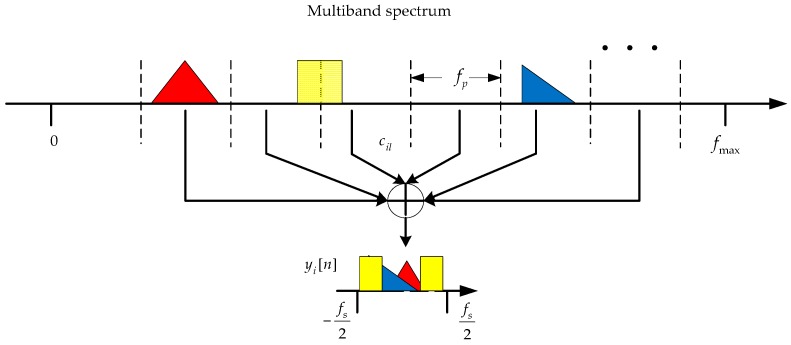
Abbreviated drawing of spectrum slices flitting.

We first review some basic knowledge about the MWC before introducing the DMWC. The MWC is a fully spectrum-blind recovery framework at the sub-Nyquist rate, which is developed from multi-coset sampling [20,21]. It uses a $T_{p}$—periodic function $p_{i}(t)$ to move all sparse sub-bands to the baseband by mixing and truncates the baseband by a LPF with cutoff frequency $f_{s}/2$, as depicted in Figure 2.

As previously mentioned, the hardware cost of sampling channels is unacceptable when the sparsity is great, because it is a necessary condition to allow accurate spectrum-blind recovery that:

(3) m≥2N .

Here m denotes the amount of channels, and N is an even number of sub-bands due to the conjugate symmetry. Besides, the input signals of all channels are completely uniform in the MWC, which is impractical in practical application.

**Figure 3:**
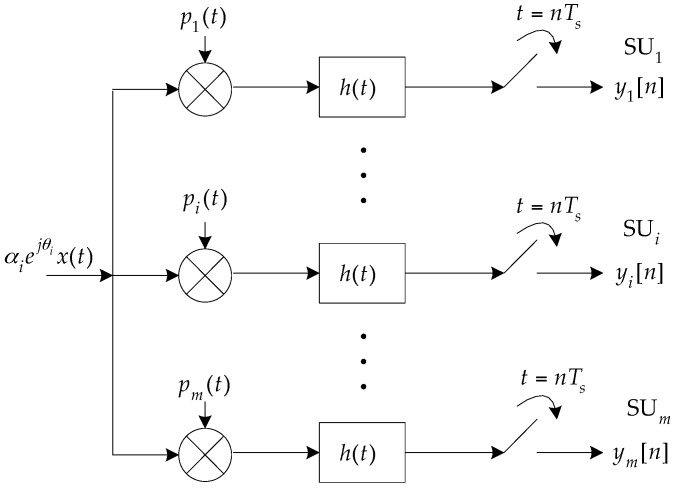
Practical DMWC sampling model.

The DMWC framework is similar to the MWC, but we take phase shift and transmission loss into account in each sampling channel, as depicted in Figure 3. The i-th SU conducts the i-th-channel sampling with a sampling interval of $T_{s}=1/f_{s}$. Here, $p_{i}(t)$ is a function that alternates between ±1 for each of the L uniform time intervals. It is expressed as:

(4) $$p_{i}(t)=B_{ik},\;k\frac{T_{p}}{L}\leq t\leq (k+1)\frac{T_{p}}{L}\;,\;0\leq k\leq L-1\;,$$

where $B_{ik}\in \{+1,-1\}$. For a periodic function, it has a Fourier expansion:

(5) $$p_{i}(t)=\sum_{l=-\infty }^{\infty }c_{il}e^{j\frac{2\pi }{T_{p}}lt}\;,$$

where

(6) $$c_{il}=\frac{1}{T_{p}}\int_{0}^{T_{p}}p_{i}(t)e^{-j\frac{2\pi }{T_{p}}lt}dt\;.$$

$B_{ik}$ alternates between ±1 chaotically, and it makes the coefficient $c_{il}$ chaotic as well.

After mixing, the Fourier transform of the analog output signal in the i-th channel can be evaluated as:

(7) $$\begin{array}{ll} {\overset{\wedge }{X}}_{i}(f) & =\int_{-\infty }^{\infty }[x_{i}(t)p_{i}(t)]e^{-j2\pi ft}dt\\ & =\int_{-\infty }^{\infty }\alpha_{i}e^{j\theta_{i}}x(t)(\sum_{l=-\infty }^{\infty }c_{il}e^{j\frac{2\pi }{T_{p}}lt})e^{-j2\pi ft}dt\\ & =\sum_{l=-\infty }^{\infty }\alpha_{i}e^{j\theta_{i}}c_{il}\int_{-\infty }^{\infty }x(t)e^{-j2\pi (f-\frac{l}{T_{p}})t}dt\\ & =\sum_{l=-\infty }^{\infty }\alpha_{i}e^{j\theta_{i}}c_{il}X(f-lf_{p}), \end{array}$$

where $f_{p}=1/T_{p}$. Similarly, the input to the LPF is a linear combination of X(f) and its $f_{p}$-shifted copies. Further, the final output sequence $y_{i}[n]$ of the i-th channel has a discrete-time Fourier transform:

(8) $$\begin{array}{ll} Y_{i}(e^{j2\pi fT_{s}}) & \;=\sum_{n=-\infty }^{\infty }y_{i}[n]e^{-j2\pi fnT_{s}}\\ & =\sum_{l=-L_{0}}^{+L_{0}}\alpha_{i}e^{j\theta_{i}}c_{il}X(f-lf_{p})\;,\;f\in [\frac{-f_{s}}{2},\frac{f_{s}}{2}]\;, \end{array}$$

where $L_{0}=\left\lceil \frac{f_{Nyquist}+f_{s}}{2f_{p}}\right\rceil -1$. Obviously, we can recover X(f) from $y_{i}[n]$ according to Equation (8). Writing Equation (8) in its matrix form, we get:

(9) $$y(f)=Az(f)\;,\;f\in [\frac{-f_{s}}{2},\frac{f_{s}}{2}]\;,$$

with the matrix, A contains its elements $a_{il}$:

(10) $$a_{il}=\alpha_{i}e^{j\theta_{i}}c_{il}^{*}\;.$$

Equation (9) builds a bridge between the DMWC and CS. Here $c_{il}$ is chaotic, as is $a_{il}$. It means A still can be considered as a random measurement matrix. Reference [14] uses a continuous-to-finite (CTF) block to recover the support S which is based on OMP. In essence, the recovery process solves the below optimization problem:

(11) $$\overset{\wedge }{z(f)}=\min{\left\|\;z(f)\;\right\|}_{0}\;s.t.\;{\left\|\;Az(f)-y(f)\;\right\|}_{2}^{2}<\eta \;,$$

where η is an acceptable error rate. Next, we analyze the influences caused by $\theta_{i}$ and $\alpha_{i}$, respectively.

### 3.1. Phase Shift $\theta_{i}$

In order to recover the original support accurately, z(f) is a jointly sparse signal that is required in the DMWC, namely $S_{i}=S$. Phase shift $\theta_{i}$ comes from a different distance $d_{i}$ between the BS and SU. More generally, we firstly assume the phase shift $\theta_{i}$ in the i-th path is a random value in [0,2π]. It is reasonably to assume that the phase shift has no effect on the support recovery while the SNR level is high enough to provide reliable reconstruction. It is not necessary to calculate $\theta_{i}$ accurately and instantly. There are two proofs to demonstrate our supposition as shown below. Firstly, $\theta_{i}$ cannot change $S_{i}$ because of the time-lapse character of the Fourier transform:

(12) $$x(t-t_{0})\overset{F}{↔}e^{-ft_{0}}X(f)\;.$$

We can see that the time shift $t_{0}$ causes no frequency shift, and the amplitude of X(f) stays the same as well. It reveals that $S_{i}$ can only be changed by $\alpha_{i}$. Secondly, atoms are selected in OMP in the recovery period by solving:

(13) $$\lambda =\arg\max\left|\langle r,\varphi_{j}\rangle \right|\;,$$

where λ denotes the index of the chosen atom, r denotes the residual and $\varphi_{j}$ is the j-th row of the measurement matrix. As a matter of course, $\theta_{i}$ has no influence on the absolute value of $\langle r,\varphi_{j}\rangle$ (whether it is a real number or a complex number). It means that we still can obtain the right index λ although we cannot get rid of $\theta_{i}$. Based on the two above-mentioned proofs, we can come to a conclusion that $\theta_{i}$ has no effect on recovering the frequency support. Moreover, it should be pointed out that such a conclusion can be true under the condition that the SNR level is high enough to provide a reliable recovery. There is no significance when the recovery is unreliable at a low SNR. Following simulations results will give a more persuasive explanation.

### 3.2. Transmission Loss $\alpha_{i}$

The transmission loss coefficient $\alpha_{i}$ of the i-th channel in the DMWC comes from the free-space path loss model. According to Equation (1), each SU can calculate $\alpha_{i}$ respectively in a CSSN. Being different from the phase shift $\theta_{i}$, $\alpha_{i}$ impairs the frequency-domain amplitude to a different extent depending on its size. On the one hand, z(f) is required to be jointly sparse. If $\alpha_{i}$ is a small value (close to 0), the power of the noise exceeds the received power $P_{a}$ potentially, with the result $S_{i}\subset S$. We assume $P_{noise}$ is the average power of the noise on the receiving terminal. For a jointly sparse property, we get:

(14) $$\alpha_{i}P_{t}>P_{noise}\;\Leftrightarrow \alpha_{i}>\frac{P_{noise}}{P_{t}}\;.$$

The lower bound of $\alpha_{i}$ can be found by Equation (14), it provides a basis for selecting suitable SUs to participate in.

Unfortunately, $\alpha_{i}$ consists of each column of A according to Equation (10). In order to reconstruct the support S with high accuracy, any two column vectors in the measurement matrix A should be non-correlated [22,23]. Now we calculate the correlation coefficient ρ of any two column vectors in Equation (10). Assuming $a_{ih}$ and $a_{ik}$ are two arbitrary column vectors of A, with 1≤i≤m, h and k are arbitrary positive integers in $\left[1,2L_{0}+1\right]$. Then we have:

(15) $$\begin{array}{ll} \rho & =\frac{\langle a_{ih},a_{ik}\rangle }{\sqrt{a_{ih}}\sqrt{a_{ik}}}\\ & =\frac{\sum_{i=1}^{m}\alpha_{i}^{2}e^{j2\theta_{i}}c_{ih}^{*}c_{ik}^{*}}{\sqrt{\sum_{i=1}^{m}\alpha_{i}^{2}e^{j2\theta_{i}}{\left(c_{ih}^{*}\right)}^{2}}\sqrt{\sum_{i=1}^{m}\alpha_{i}^{2}e^{j2\theta_{i}}{\left(c_{ik}^{*}\right)}^{2}}}\;. \end{array}$$

Because $\alpha_{i}\in (0,1)$, when $\alpha_{i}$ becomes close to1 infinitely, the DMWC has the same ρ as the MWC. In this case, ρ approaches 0 as $c_{il}$ is chaotic. It means that each channel has no transmission loss (situation in the MWC); thus, the DMWC will have the same recovery performance as the MWC theoretically. On the contrary, when $\alpha_{i}$ becomes close to 0 infinitely, it leads to $a_{ih}$ and $a_{ik}$ both being zero vectors. It means ρ approaches 1, because the two near-equal vectors have a maximal correlation. Accordingly, it is impossible to recover S when $\alpha_{i}$ becomes close to 0. Based on the above-mentioned analysis, the absolute value of ρ is increasing as $\alpha_{i}$ decreases, namely the non-correlation between $a_{ih}$ and $a_{ik}$ is weakened. Although we cannot prove the increasing/decreasing property of Equation (15) strictly, we can draw a conclusion from the results of numerical simulations. The recovery accuracy reduces as $\alpha_{i}$ decreases, which will be verified in the numerical simulations section.

### 3.3. Three-Time Handshake Mechanism

Here we propose a three-time handshake mechanism to coordinate the communication between the FC and SUs within a one-spectrum sensing cycle. The mechanism is described as:

$$\begin{array}{l} \mathrm{START}\\ ⇓\\ \mathrm{First}\;\mathrm{handshake}\;\{\begin{array}{l} \;\mathrm{FC}\;\mathrm{selects}\;\mathrm{suitable}\;\mathrm{SUs},\;\mathrm{broadcasts}\;\mathrm{sensing}\;\mathrm{task}.\\ \mathrm{Selected}\;\mathrm{SUs}\;\mathrm{confirm}\;\mathrm{to}\;\mathrm{join}\;\mathrm{in},\;\mathrm{each}\;\mathrm{SU}\;\mathrm{conducts}\;\mathrm{one}\;\mathrm{sampling}\;\mathrm{channel}. \end{array}\\ ⇓\\ \mathrm{Second}\;\mathrm{handshake}\{\begin{array}{l} \;{\mathrm{SU}}_{i}\;\mathrm{transmits}\;\mathrm{sampling}\;\mathrm{sequence}\;y_{i}[n]\;\mathrm{to}\;\mathrm{FC}.\\ \mathrm{FC}\;\mathrm{collects}\;\mathrm{sampling}\;\mathrm{data}\;\mathrm{and}\;\mathrm{uses}\;\mathrm{CTF}\;\mathrm{to}\;\mathrm{recover}\;S,\;\mathrm{broadcasts}\;\mathrm{sensing}\;\mathrm{result}. \end{array}\\ ⇓\\ \mathrm{Third}\;\mathrm{handshake}\;\{\begin{array}{l} {\mathrm{SU}}_{i}\;\mathrm{applys}\;\mathrm{to}\;\mathrm{FC}\;\mathrm{for}\;\mathrm{accessing}\;\mathrm{to}\;\mathrm{spectrum}\;\mathrm{holes}\;\mathrm{according}\;\mathrm{to}\;S.\\ \mathrm{FC}\;\mathrm{allocates}\;\mathrm{spectrum}\;\mathrm{holes},\;\mathrm{allows}\;\mathrm{the}\;\mathrm{access}\;\mathrm{requests}.\; \end{array}\\ ⇓\\ \mathrm{END} \end{array}$$

This mechanism aims at ensuring the communication synchronization between the FC and SUs, which can avoid compromising the quality of service (QoS) of the authorized channels. It is worth mentioning that the sub-Nyquist sampling sequence $y_{i}[n]$ transmitted in the CSSN makes a big contribution to the data security because of the pseudo-randomness of A.

## 4. Numerical Simulations and Discussion

Numerical simulations are conducted in this section to evaluate the recovery performance of the DMWC. The results will give guidance to select cooperative SUs reasonably. The probability of exact detection $P_{d}$ is an important factor in the spectrum sensing field. Here we calculate the probability of exact support recovery $P_{r}$ by 1000 Monte Carlo simulations (MCs). Necessarily, if the frequency support S is recovered exactly, the spectrum holes are located reliably. It indicates that:

(16) $$P_{d}=P_{r}\;.$$

The simulation signal model is given by:

(17) $$x(t)=\sum_{i=1}^{N/2}\sqrt{E_{i}B}\;\mathrm{sinc}(Bt)\cos(2\pi f_{i}t)\;,$$

where each width B equals 50 MHz. The energy coefficient $E_{i}$ is random in (0,10], and the carrier $f_{i}$ is random within [0,5 GHz]. It means that we need a Nyquist sampling rate $f_{Nyquist}$ equal to 10 GHz. In addition, $f_{s}\geq f_{p}$ is a necessary condition to allow accurate spectrum-blind recovery according to [14]. We make the smallest $f_{s}=f_{p}=f_{Nyquist}/L$, where L=195 is the length of $p_{i}(t)$. So the compressive ratio (CR) in a single sampling channel is $f_{s}/f_{Nyquist}=1/L$, and the CR of the DMWC system (all channels) is m/L. The bigger m is, the greater the CR is. It can make a tradeoff between $P_{r}$ and the CR, depending on our practical demand.

### 4.1. Support Recovery with Random $\theta_{i}$

**Figure 4:**
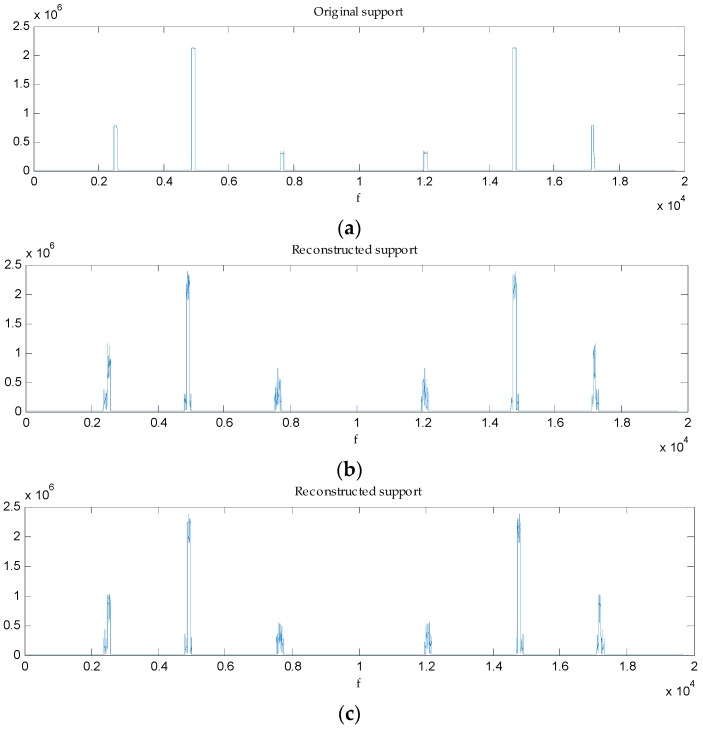
Frequency support recovery when N=6. (**a**) Original support; (**b**) Reconstructed support when no $\theta_{i}$ exists in the path; (**c**) Reconstructed support when random $\theta_{i}$ exists in the path.

**Figure 5:**
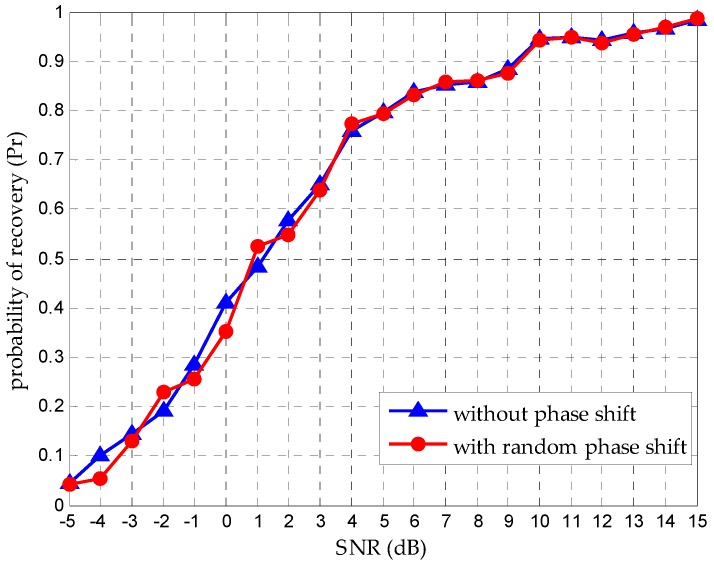
Comparison of $P_{r}$ under random phase shift and no phase shift when N=6.

It is important to emphasize that what we care about in spectrum sensing is the accurate frequency support recovery, not the time-domain expression. We have a conclusion that random phase shift has no effect on the support recovery under the condition the SNR level is high enough to provide a reliable recovery. We evaluate the recovery performance when random $\theta_{i}$ exists in each path. As Figure 4 shows, the random phase shift makes no change to support recovery on a reliable reconstruction. We verify this conclusion at different SNR levels, as Figure 5 shows. It makes clear that support recovery performances have a high degree of consistency when the SNR is above 5 dB, which is in accordance with our analysis in Section 3.1. When the SNR is below 5 dB, there is a discrepancy because of the recovery unreliability.

### 4.2. Seeking an Acceptable $\alpha_{i}$

**Figure 6:**
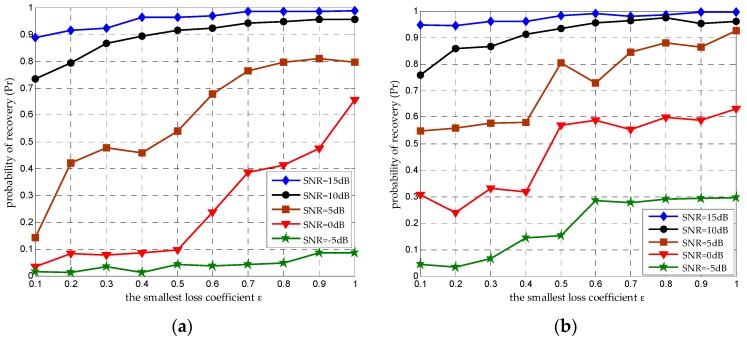
The curvilinear relation between ε and $P_{r}$ under different SNR levels, when the amount of sub-bands is N=6 and the number of cooperative SUs is m=50. Simulations results are presented for (**a**) CR = 0.25 and (**b**) CR = 0.5.

As indicated earlier, the recovery accuracy will reduce as $\alpha_{i}$ decreases, because the non-correlation between $a_{ih}$ and $a_{ik}$ is weakened. The DMWC must seek an acceptable $\alpha_{i}$ value, which is its important contribution in practical engineering. We assume the smallest $\alpha_{i}$ in all channels is denoted by ε, namely $\alpha_{i}\in [\varepsilon ,1)$. There is a curvilinear relation between ε and $P_{r}$, as Figure 6 depicts. We can find that the recovery performance improves smoothly as ε increases at a high SNR level. However, when the SNR is set to 0 dB and 5 dB, the recovery performance has a wide fluctuation under different ε. Worse still, when the SNR is below 0 dB, the performance is terrible with all ε. We can give an explanation from two aspects: on the one hand, OMP can ensure the exact support recovery under the condition that the SNR must at least scale linearly with the sparsity of the broadband signal [24]. In our simulations, we set the amount of sub-bands N=6. SNR ≥ 10 dB can ensure exact support recovery with high probability, while 0 dB and 5 dB work worse, and being below 0 dB is unacceptable. On the other hand, if ε is much too small, it will badly damage the jointly sparse property of all SUs so that we cannot achieve the exact support recovery.

In fact, it is difficult to give a closed-form expression between ε and $P_{r}$. We can only define a suitable ε according to a desired $P_{r}$ under different SNR levels. When ε=0.8, we can get a $P_{r}$ roughly equal to 0.95 when the SNR is 10 dB. Once ε is fixed, the longest distance $d_{\max}$ is confirmed by Equation (1), and all m cooperative SUs must be selected within $d_{\max}$. Such a message is extremely significant for a CSSN to select SUs as its sensor nodes.

### 4.3. Increasing m to Improve $P_{r}$

**Figure 7:**
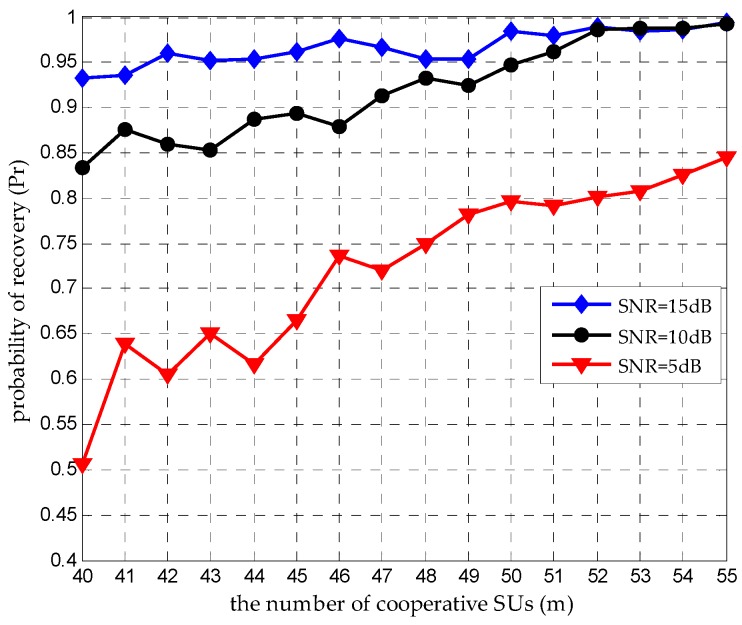
The relation between m and $P_{r}$ under different SNR levels when N=6 and ε=0.8.

**Figure 8:**
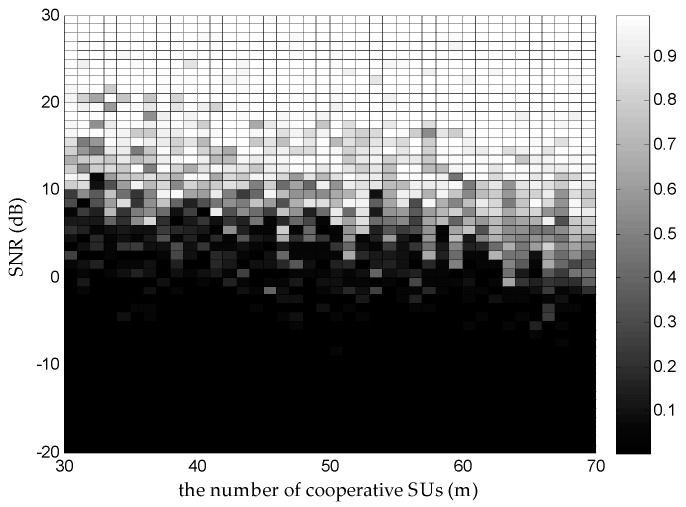
Image intensity denotes the value of $P_{r}$ under the different m and different SNR levels when ε=0.8.

According to Equation (1), $\alpha_{i}$ is determined by $d_{i}$. DMWC cannot keep away from this adverse factor. For a measurement matrix A, which is of size m×M, it has the restricted isometry property (RIP) of order K if m≥CKlog(M/K), where K denotes the sparsity and C is a positive constant [25]. It indicates that when m is greater than CKlog(M/K), A has a higher probability to satisfy the RIP. In [14], when N=6, it makes C=4 to get a stable recovery; then m≈50. We change m and evaluate $P_{r}$ at different SNR levels, as Figure 7 shows. When m is above 49, $P_{r}$ improves steadily as m increases at a high SNR. In other words, m≥4Nlog(M/2N) can be a ruler, which determines the amount of cooperative SUs in a CSSN. Besides, we should choose m reasonably under different ε, for the sake of getting a desired $P_{r}$. Figure 8 reports more results under different m and SNR levels. Compared with the result in [14] (ε=1 in the MWC situation), we can find that the DMWC is more sensitive to noise levels because noise energy and $\alpha_{i}$ damage the jointly sparse property of all SUs together. However, it is convenient to add SUs as sampling channels in the DMWC, which benefits from node-based sensing turning into network-based sensing.

### 4.4. Time-Varying Support Problem

**Figure 9:**
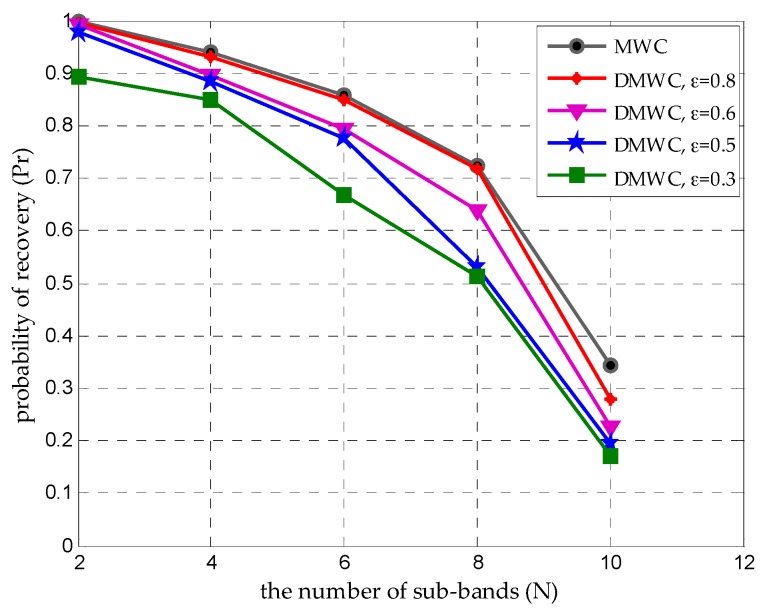
Probability of support recovery under time-varying S at different ε when m=50.

When frequency support S is time-varying, it means the number of sub-bands and the positions of sub-bands are time-varying. According to the necessary condition to allow exact recovery, the amount of sampling channels will increase proportionally as N becomes greater. Nevertheless, the DMWC can add channels dynamically and flexibly by adding cooperative SUs to reply to the time-varying support problem. We evaluate $P_{r}$ under a time-varying N at different ε. Figure 9 reports the comparison between the MWC and DMWC. It indicates the DMWC has a worse performance than the MWC when m is constant because of the transmission loss $\alpha_{i}$, especially at low ε levels. The good news is that we can easily design a large enough value as the initial m to deal with the time-varying support.

## 5. Conclusions

In this paper, we propose an original DMWC scheme. DMWC treats a single geographical sensor node as a sampling channel, turns node-based sensing into network-based sensing, and reduces the hardware cost drastically for a single node. Theoretical analysis and numerical simulations indicate that phase shift has no influence on the recovery of frequency support in the DMWC. However, transmission loss degrades the recovery performance to a different extent. In order to get a desired sensing performance, we explore how to choose cooperative SUs reasonably according to the transmission distance in a CSSN. Finally, we have a conclusion that we can improve the recovery performance by increasing the amount of cooperative SUs dynamically and flexibly. Although we can design a large initial m to deal with the time-varying support problem, to improve the recovery performance in a bad transmission attenuation situation and adjust the number of cooperative SUs automatically, we need further research.
